# The Roles of PCSK9 in Alzheimer’s Disease: A Systematic Review of Clinical, Genetic, and Preclinical Evidence

**DOI:** 10.3390/life15121851

**Published:** 2025-12-02

**Authors:** Vicko Suswidiantoro, Meidi Utami Puteri, Mitsuyasu Kato, Donna Maretta Ariestanti, Richard Johari James, Fadlina Chany Saputri

**Affiliations:** 1 Doctoral Programme, Faculty of Pharmacy, Universitas Indonesia, Kota Depok 16424, Indonesia; 2 Laboratory of Pharmacology and Toxicology, Faculty of Pharmacy, Universitas Indonesia, Kota Depok 16424, Indonesia; 3 National Metabolomics Collaborative Research Centre, Faculty of Pharmacy, Universitas Indonesia, Kota Depok 16424, Indonesia; 4 Faculty of Pharmacy, Universitas Indonesia, Kota Depok 16424, Indonesia; 5 Department of Experimental Pathology, Faculty of Medicine, University of Tsukuba, Tsukuba 305-8575, Ibaraki, Japan; 6 Division of Cell Dynamics, Transborder Medical Research Center, University of Tsukuba, Tsukuba 305-8575, Ibaraki, Japan; 7 Integrative Pharmacogenomics Institute (iPROMISE), UiTM Selangor Branch, Puncak Alam Campus, Bandar Puncak Alam 42300, Selangor, Malaysia; 8 Faculty of Pharmacy, UiTM Selangor Branch, Puncak Alam Campus, Bandar Puncak Alam 42300, Selangor, Malaysia

**Keywords:** Alzheimer’s disease, amyloid-β, cholesterol, neurodegeneration, PCSK9

## Abstract

Alzheimer’s disease (AD) is increasingly associated with alterations in cholesterol metabolism. Proprotein convertase subtilisin/kexin type 9 (PCSK9), an enzyme regulating low-density lipoprotein receptor (LDLR) degradation, has been implicated in AD through mechanisms involving amyloid-β (Aβ) processing, tau phosphorylation, and synaptic dysfunction. This review aimed to evaluate clinical, genetic, and experimental evidence regarding the role of PCSK9 in AD and its potential as a biomarker or therapeutic target. A systematic search was conducted in PubMed, Scopus, ScienceDirect, and Google Scholar (2020–2025) using predefined terms related to PCSK9 and Alzheimer’s disease. Eligible studies included clinical, in vivo, and in vitro investigations reporting PCSK9 expression, regulation, or inhibition in relation to AD pathology. Due to methodological heterogeneity, a narrative synthesis was performed. Forty-two studies met inclusion criteria. Preclinical findings consistently showed that elevated PCSK9 may indirectly promote Aβ accumulation, tau hyperphosphorylation, neuroinflammation, and cognitive decline, while genetic deletion or pharmacological inhibition of PCSK9 mitigates these effects. Clinical evidence was variable: several studies identified increased PCSK9 levels in cerebrospinal fluid or brain tissue of AD patients, often correlating with tau markers, but large-scale genetic and Mendelian randomization studies did not confirm a causal association. PCSK9 inhibitors, widely used in cardiovascular therapy, demonstrated potent LDL-C reduction without cognitive adverse effects. Experimental data suggest that PCSK9 contributes to AD-related pathology, whereas human evidence indicates a modulatory or biomarker role rather than a causative one. Despite strong preclinical data, human genetics lacks causal evidence for PCSK9 in Alzheimer’s. It may be a disease modifier or biomarker; its clinical relevance requires confirmation through longitudinal studies and CNS-penetrant therapies.

## 1. Introduction

Over recent decades, increasing attention has focused on the role of cholesterol metabolism in AD pathogenesis [1]. Cholesterol is indispensable for neuronal membranes, where it regulates fluidity, synapse formation, and synaptic signaling. In the brain, it is synthesized locally, predominantly by astrocytes and neurons, under strict homeostatic control [2]. Epidemiological studies consistently report that midlife hypercholesterolemia increases the risk of developing AD later in life [3,4]. One proposed mechanism involves amyloid precursor protein (APP) processing: cholesterol-rich lipid rafts are thought to facilitate β- and γ-secretase activity, enhancing the generation of the pathogenic Aβ42 peptide [5]. In addition, cholesterol influences both Aβ aggregation and its clearance.

Proprotein convertase subtilisin/kexin type 9 (PCSK9) has emerged as a key regulator of systemic cholesterol homeostasis [6]. This secreted protease, produced mainly in the liver, binds to low-density lipoprotein receptors (LDLRs) on hepatocytes, promoting their lysosomal degradation. By reducing receptor availability, PCSK9 elevates circulating LDL-C levels [7,8]. Gain-of-function mutations in PCSK9 result in familial hypercholesterolemia, whereas loss-of-function variants confer lower cholesterol levels and a reduced risk of cardiovascular disease [9]. These discoveries paved the way for the development of PCSK9 inhibitors such as alirocumab and evolocumab capable of lowering LDL-C [10,11]

Importantly, PCSK9, first described as neural apoptosis-regulated convertase 1 (NARC-1), has been recognized as a distinct member of the proprotein convertase enzyme family [12]. Since its identification in the regulation of cholesterol homeostasis, a substantial number of investigations have explored its relationship with cardiovascular disorders and their contributing risk elements. Emerging studies highlight that PCSK9 is a pivotal modulator of plasma cholesterol concentration, primarily through facilitating the breakdown of low-density lipoprotein receptors (LDLR) [13,14]. PCSK9 is also expressed within the central nervous system, including the cortex, hippocampus, and cerebellum, where it is detected in neurons, astrocytes, and ependymal cells [15,16]. Although its precise role in the brain remains incompletely understood, PCSK9 is thought to regulate neuronal surface receptors involved in lipid handling, signaling, and survival [17]. In vitro evidence indicates that PCSK9 negatively regulates key receptors like LDLR, LRP1, and ApoER2, which are essential for cerebral cholesterol homeostasis and ApoE metabolism. Given the central role of ApoE as the brain’s main lipid carrier and its established genetic link to AD, the influence of PCSK9 on Alzheimer’s pathology is probably indirect. This effect is mediated by disrupting the processes of Aβ clearance and synaptic function, not via a direct pathway [16,18]. Excess PCSK9 in the brain may therefore hinder Aβ removal, exacerbating Aβ pathology and linking peripheral dyslipidemia to central AD mechanisms [15,18].

Scientific interest in the role of PCSK9 in Alzheimer’s disease has led to a wide range of research approaches. Researchers have explored whether certain PCSK9 gene variants are linked to a higher likelihood of developing Alzheimer’s, measured PCSK9 levels in the blood and cerebrospinal fluid of patients, and carried out experimental studies to see what happens when PCSK9 is inhibited [19,20,21]. Beyond these basic and preclinical findings, large cardiovascular trials involving PCSK9 inhibitors have provided an additional layer of insight. Long-term safety data and follow-up analyses from these studies have offered early hints of a possible connection between PCSK9 and dementia risk [11,22].

Nevertheless, findings remain inconsistent and at times contradictory. Some genetic studies suggest that PCSK9 loss-of-function variants confer protection against AD, whereas others show no association. Similarly, preclinical work alternately supports pro-amyloidogenic effects, negligible impact, or even context-dependent neuroprotection. This heterogeneity highlights the need for a comprehensive and critical synthesis of the available evidence [20,23].

## 2. Materials and Methods

### 2.1. Search Strategy

This review follows the Preferred Reporting Items for Systematic Reviews and Meta-Analyses (PRISMA). A comprehensive search strategy was applied across PubMed/MEDLINE, Scopus, ScienceDirect and Google Scholar 2020–2025. The search combined controlled vocabulary terms (MeSH/Emtree) and free-text keywords for “PCSK9” and “Alzheimer’s disease,” with syntax adapted for each database. For PubMed, an example search string was (“PCSK9” OR “PCSK9 protein, human” OR “Proprotein Convertase 9” OR “Proprotein Convertase Subtilisin/Kexin Type 9”) AND (“Alzheimer’s Disease” OR “Dementia, Alzheimer’s Type” OR “Cognitive Dysfunction” OR “Amyloid-β Peptides” OR tau OR “Neurofibrillary Tangles”). Gray literature sources and the reference lists of included articles and related reviews were also scrutinized to identify additional relevant studies.

### 2.2. Eligibility Criteria

We included studies that examined any potential role of PCSK9 in Alzheimer’s disease, spanning clinical, genetic, in vivo, and in vitro approaches. Clinical evidence covered observational cohorts, randomized controlled trials, and genetic analyses such as Genome-wide association studies (GWAS) and Mendelian randomization, provided they reported outcomes relevant to cognition, Alzheimer’s biomarkers, or disease risk. Experimental studies in animal models or cell-based systems were considered if they explored PCSK9 expression, manipulation, or inhibition in relation to Aβ deposition, tau pathology, inflammation, or neuronal survival. Only peer-reviewed articles published in English were eligible, while reviews, conference proceedings, and reports without primary data were excluded.

### 2.3. Study Selection and Data Extraction

Following duplicate removal, the remaining records were screened by title and abstract. Full texts of potentially relevant studies were then reviewed in detail. Any disagreements about inclusion were resolved by consensus among reviewers. Data were collected systematically, covering study characteristics (population or model, intervention or exposure, outcome measures) as well as the key findings. These data were subsequently organized into structured tables: clinical evidence, animal studies, and cell culture experiments.

### 2.4. Data Synthesis

Due to the heterogeneity in methodologies, populations, and outcome measures among the included studies, a meta-analysis was not deemed appropriate. Instead, a narrative synthesis was performed. Findings were categorized and analyzed according to study type: clinical (including genetic and trial data), in vivo, and in vitro. Key results from each investigation were systematically extracted and compiled into evidence tables to facilitate comparison across studies. The synthesis aimed to identify consensus patterns, explore mechanistic pathways, and critically evaluate points of divergence among the different research approaches to provide a comprehensive assessment of PCSK9’s role in AD. Possible sources of heterogeneity were evaluated along three principal dimensions: study design (clinical investigations, in vivo experiments, or in vitro studies); experimental context (human, animal, or cell-based models, and the specific type of PCSK9 modulation—such as inhibition, overexpression, or knockout); and outcome categories (including Aβ accumulation, tau phosphorylation, neuroinflammatory markers, lipid regulation, and cognitive outcomes).

To assess robustness, we examined whether the overall conclusions were affected by: (1) excluding studies with insufficient methodological information or uncertain Alzheimer’s disease diagnoses; (2) assigning greater interpretive value to investigations with larger cohorts, validated transgenic models (e.g., 5xFAD, APP/PS1), or well-controlled experimental settings; and (3) evaluating the consistency of findings across different research groups and publication periods.

After these qualitative evaluations, the overarching patterns—particularly the direction and nature of PCSK9’s impact on Aβ, tau, and neuroinflammatory processes—remained stable, reinforcing the reliability of the synthesized conclusions.

## 3. Results

### 3.1. Study Selection

**Figure 1:**
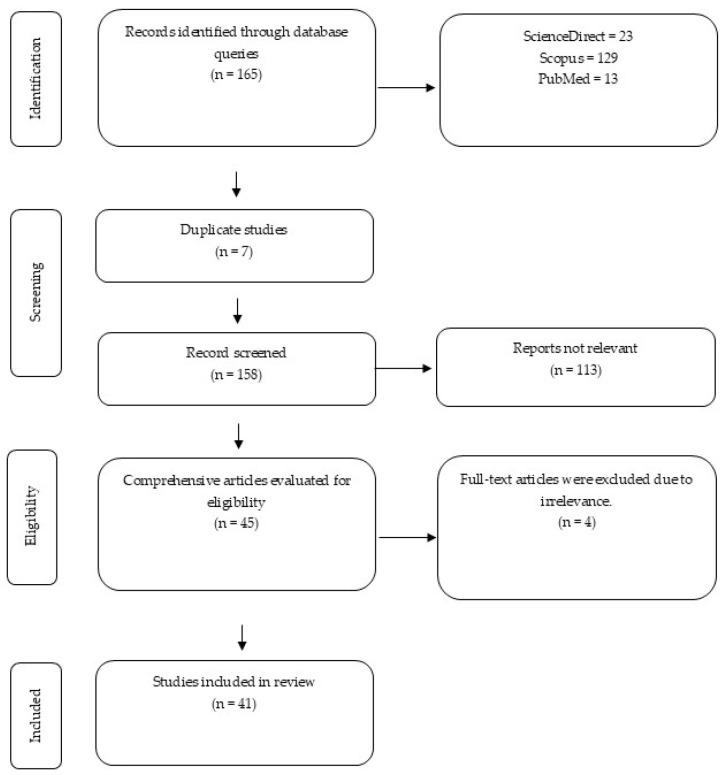
The diagram outlines the identification, screening, eligibility, and inclusion stages of the systematic review.

The literature search identified 165 records. After removing seven duplicates, 158 unique studies were screened. Of these, 113 were excluded based on titles and abstracts, leaving 45 articles for full-text assessment. Four did not meet the eligibility criteria after full review, resulting in 41 studies being included in the final synthesis Figure 1.

### 3.2. Clinical Evidence

As summarized in Table 1, results from 20 clinical investigations were mixed. Several studies reported elevated PCSK9 levels in cerebrospinal fluid or brain tissue from patients with Alzheimer’s, often in parallel with increased tau or phosphorylated tau [15,19,24]. By contrast, genetic analyses—including GWAS and Mendelian randomization—generally failed to identify a causal link between PCSK9 and disease risk [23,25,26]. Trials of PCSK9 inhibitors in cardiovascular populations consistently showed substantial reductions in LDL cholesterol without negative cognitive effects [11,22]. Some smaller studies even suggested potential cognitive improvements, though these findings remain preliminary. Importantly, variability across clinical data appears influenced by sex, APOE genotype, and vascular comorbidities [15,27]. Taken together, clinical findings point to a possible role of PCSK9 as a biomarker of neurodegeneration rather than as a direct driver of Alzheimer’s risk. Although initial genetic research pointed to a potential link, more recent and extensive genetic studies have not supported a causal relationship between PCSK9 and AD. For example, a 2023 Mendelian randomization study by Larsson et al. found no genetic evidence that inhibiting PCSK9 affects AD risk [28]. This conclusion is reinforced by a 2025 GWAS meta-analysis from Wu et al., which also did not identify PCSK9 as a significant genetic risk factor for Alzheimer’s. Together, these findings suggest that earlier associations observed in clinical cohorts are likely correlational, not causative [29]. The relationship between PCSK9 and AD appears to be one of influence rather than direct causation [30]. On one hand, the concentration of PCSK9 in a patient’s cerebrospinal fluid or bloodstream may fluctuate with the disease’s progression and tau pathology, suggesting it could be a useful dynamic biomarker. However, extensive human genetic data currently lacks the evidence to classify PCSK9 as a direct causal factor in the development of the disease.

### 3.3. In Vivo Evidence

The animal studies reviewed in the Table 2 provided stronger and more consistent results. Overexpression of PCSK9 in transgenic mice accelerated Aβ build-up, promoted tau phosphorylation, and impaired memory performance [21,31]. Research indicates that PCSK9-mediated internalization and lysosomal degradation of key receptors (LDLR, LRP1, ApoER2) reduce their presence on the neuronal surface. This loss impairs critical cellular functions, namely cholesterol import and the clearance of Aβ. The resulting dysregulation of cholesterol homeostasis, particularly within lysosomes and lipid rafts, creates conditions that boost the function of β- and γ-secretases. This elevated secretase activity drives the amyloidogenic processing of the APP, thereby accelerating the deposition of Aβ. Observations from studies using APP/PS1 and 5xFAD rodent models consistently support this pathogenic sequence [32]. Conversely, knockout models or treatment with PCSK9 inhibitors alleviated pathology, enhanced synaptic function, and preserved cognition [33,34]. Several studies also highlighted systemic effects, showing that PCSK9 suppression improved vascular integrity, reduced oxidative stress, and dampened neuroinflammatory responses [35,36]. These findings suggest that PCSK9 contributes to AD pathology not only through neuronal mechanisms but also by worsening vascular and metabolic dysfunction.

### 3.4. In Vitro Evidence

Cellular models have provided important mechanistic insights into how PCSK9 may contribute to AD pathology in the Table 3. Studies using neuronal and glial cultures indicate two primary effects: (1) reduction in lipoprotein receptor availability on the cell surface (including LDLR, LRP1, and ApoER2), leading to impaired cholesterol uptake and diminished clearance of Aβ; and (2) modulation of APP processing, favoring the amyloidogenic pathway and thereby increasing Aβ production. These findings align with in vivo observations that elevated PCSK9 levels exacerbate Aβ accumulation and synaptic dysfunction [16,37]. At the molecular level, overexpression or exogenous addition of PCSK9 has been shown to accelerate the internalization and lysosomal degradation of LDLR-family receptors. This process decreases intracellular cholesterol availability and alters the lipid raft environment, conditions that are known to enhance β- and γ-secretase activity toward APP, ultimately increasing production of the neurotoxic Aβ42 peptide. In addition, PCSK9’s interactions with membrane proteins such as APLP2 and LRP1 have been proposed as potential mechanisms that redirect receptor trafficking and APP processing [37,38]. It is crucial to recognize that the biological action of PCSK9 is not consistent across all conditions. Paradoxically, inhibiting PCSK9 has been shown in some models to elevate Aβ in the brain, likely by disrupting the management and lipidation of ApoE isoforms. This finding reveals the complex nature of the interaction between cholesterol and Aβ pathology.

**Table 1:** Summary of Clinical Studies on PCSK9 and AD.

Author (Year)	Population/Model	Intervention/Methods	Main Findings
Bejaoui et al. (2025) [39]	14,669 healthy individuals	DNA methylation & sequencing	APOE * E2 and PCSK9 variants are protective Associated with delayed aging Immune and cardiovascular modulation observed
Benn et al. (2017) [23]	>100,000 human cohorts (Denmark/UK)	Genetic proxies (MR	PCSK9/HMGCR variants ↓ LDL-C Reduced cardiovascular risk Increased diabetes risk No association with dementia or AD
Caselii et al. (2019) [40]	539 suspected CAD (EVINCI)	Plasma PCSK9 + coronary CTA	Low PCSK9 linked to metabolic syndrome Associated with obesity and insulin resistance Related to diffuse coronary artery disease
Courtemanche et al. (2018) [19]	67 patients (36 AD, 31 controls)	CSF PCSK9,	CSF PCSK9 elevated in neurodegeneration Correlates with AD biomarkers Aβ and tau
Giugliano et al. (2017) [22]	1204 ASCVD patients	Evolocumab vs. placebo (EBBINGHAUS RCT)	No cognitive differences between groups Confirms cognitive safety of PCSK9 inhibition
Harvey et al. (2018) [41]	Statin/PCSK9i users (multinational)	RCT meta-analysis	No increase in neurocognitive adverse events
Huang et al. (2024) [26]	462,933 participants (China/UK Biobank)	Mendelian randomization	PCSK9 inhibition ↓ LDL-C and ↓ CVD No association with dementia or depression
Korthauer et al. (2022) [42]	868 elderly (65–83y, Germany)	Cognitive tests + APOE genotyping	No association of APOE genotype, lipid-lowering, cognition
Lambert et al. (2013) [25]	74,046 individuals (I-GAP)	GWAS meta-analysis	Identified 19 AD-associated loci beyond APOE
Lee et al. (2025) [43]	117 BD-II patients + 41 controls	Plasma PCSK9 biomarkers	PCSK9 correlated with cognition in controls No correlation in BD-II patients
Lütjohann et al. (2021) [44]	28 hypercholesterolemic patients	Alirocumab/evolocumab	↓ Cholesterol and ↓ oxysterols Altered brain cholesterol metabolism
Paquette et al. (2018) [20]	878 (468 AD cases, 410 ctrls, Canada)	PCSK9 LOF genotyping	No association with AD risk or cognition
Picard et al. (2019) [15]	AD brains + controls + at-risk individuals	Protein/mRNA/eQTL	PCSK9 elevated in AD brain and CSF Female-specific PCSK9 risk variants identified
Postmus et al. (2013) [45]	5777 elderly (PROSPER)	PCSK9 SNP rs11591147	Variant ↓ LDL-C No effect on cognition or daily functioning
Robinson et al. (2015) [10]	2341 high-risk patients (ODYSSEY)	Alirocumab vs. placebo	↓ LDL-C by ~62% ↓ Major cardiovascular events Mild neurocognitive events, reversible
Rosoff et al. (2022) [46]	~740,000 European ancestry	MR study	PCSK9 inhibition cognitively neutral Small adverse cognitive signal for HMGCR
Sabatine et al. (2017) [11]	27,564 ASCVD (FOURIER)	Evolocumab vs. placebo	↓ LDL-C by ~59% ↓ Major cardiovascular events No cognitive harm
Seijas-Amigo et al. (2023) [47]	158 ASCVD/familial hyperchol. (Spain)	Alirocumab/evolocumab (24mo MEMOGAL)	No cognitive impairment; small memory improvement
Shahid et al. (2022) [48]	Systematic review (20 trials)	Statins vs. PCSK9 inhibitors	Rare, reversible cognitive dysfunction Large RCTs consistently show safety
Simeone et al. (2021) [27]	166 high CV-risk (Italy)	Plasma PCSK9 + cognitive tests	PCSK9 linked to memory in females Indicates sex-specific risk
Xu et al. (2014) [49]	281 CAD patients (China)	Plasma PCSK9 + lipoprotein subfractions	PCSK9 associated with LDL size/atherogenic score Stronger associations in men
Zimetti et al. (2016) [24]	30 AD vs. 30 controls (Italy)	CSF PCSK9 + ApoE	CSF PCSK9 elevated in AD Correlated with ApoE4 and tau levels

* = refers to the E2 allele of the APOE gene or the likes ε as nomenclature. ↓ = shows the reduced.

**Table 2:** Summary of In vivo Studies on PCSK9 and AD.

Author (Year)	Population/Model	Intervention	Main Findings
Abuelezz et al. (2021) [34]	Wistar rats + HFCD	Alirocumab vs. memantin	Improved cognition ↓ Aβ42 levels Restored cholesterol homeostasis
Apaijai et al. (2019) [50]	52 Wistar rats (cardiac I/R model)	PCSK9 inhibitor (10 µg/kg IV)	↓ Brain inflammation ↓ Brain Aβ accumulation Preserved dendritic spine density
Arunsak et al. (2020) [35]	Sprague-Dawley rats + HFD	PCSK9 inhibitor vs. atorvastatin	Improved spatial learning & memory ↓ Aβ deposition ↓ Neuroinflammation
Dong et al. (2023) [51]	APP/PS1 mice + rat neurons	Resveratrol, suramin, siRNA	Aβ increased PCSK9 & ApoE, reduced LDLR Resveratrol reversed changes via SIRT1–PGC1α pathway
Grames et al. (2018) [21]	APP/PS1 mice	AV8-PCSK9 gene transfer	PCSK9 overexpression → hypercholesterolemia Modest increase in amyloid burden
Hernandez Torres et al. (2024) [31]	PCSK9DY mice (C57BL/6N)	AAV-PCSK9DY + Western diet	↑ BBB leakage ↓ Cerebral blood flow (CBF) Impaired memory performance
Kysenius et al. (2012) [52]	Mouse neurons (CGN & DRG)	PCSK9 RNAi knockdown	Knockdown-protected neurons via ApoER2 upregulation
Liu et al. (2010) [32]	WT, KO & TG mice	Genetic manipulation	PCSK9 bound LDLR/VLDLR/ApoER2 No detectable CNS functional effect
Mazura et al. (2022) [18]	5xFAD mice + BBB cells	PCSK9, anti-PCSK9 mAbs	PCSK9 reduced Aβ clearance across BBB Anti-PCSK9 improved memory
Shabir et al. (2022) [53]	PCSK9-ATH & J20-AD mice	AAV-PCSK9-induced atherosclerosis	PCSK9-ATH impaired neurovascular coupling
Viella et al. (2024) [33]	5xFAD mice + astrocytes	PCSK9 knockout + Aβ exposure	Improved memory function ↓ Amyloid burden Modulated neuroinflammation
Wagner et al. (2024) [36]	Rats, chronic ethanol	Alirocumab	↑ LDLR expression ↓ Oxidative stress Preserved BBB integrity ↓ Neuronal damage
Yang et al. (2024) [54]	Streptozotocin-induced T2DM rats	PCSK9 inhibitor	↓ Inflammation ↑ LDLR expression Improved cognitive function
Zhao et al. (2017) [55]	ApoE(−/−) mice + HFD	Diet-induced hyperlipidemia	↑ PCSK9 and BACE1 expression Amyloid plaque formation Neuronal apoptosis

↓ = shows the reduced. ↑ = shows the increased/elevated.

**Table 3:** Summary of In vitro Studies on PCSK9 and AD.

Author (Year)	Population/Model	Intervention	Main Findings
DeVay et al. (2013) [56]	Human hepatocyte-derived cells (HepG2, Huh7, HEK293)	PCSK9, antibodies, siRNA	APLP2 directs PCSK9 to lysosomes Promotes lysosomal degradation of LDLR
Fu et al. (2017) [57]	CHO cells, mouse neurons	siRNA, overexpression	PCSK9 interacts with APP/APLP2/LRP1, not essential for degradation
Kysenius et al. (2016) [38]	Primary cerebellar granule neurons (CGNs)	Primary cerebellar granule neurons (CGNs)	Stressors increase VLDLR expression Alter Wnt/β-catenin signaling Activate Alzheimer’s-related pathway
Papotti et al. (2022) [37]	Astrocytes & neurons (U373 + Aβ fibrils)	PCSK9 treatment	Reduces cellular cholesterol Decreases LDLR and ApoER2 expression Exacerbates Aβ toxicity
Rousselete et al. (2011) [16]	Mouse primary neurons	PCSK9 overexpression/knockdown	PCSK9 ↓ LDLR, impaired cholesterol uptake

↓ = shows the reduced.

## 4. Discussion

The possible role of PCSK9 in AD has attracted growing interest, but the data remain inconsistent and sometimes conflicting. Experimental work in cells and animal models strongly supports the view that PCSK9 promotes pathological changes relevant to AD, including Aβ deposition, tau abnormalities, and neuronal death. Clinical investigations, however, have not always reproduced these associations, and in many cases, the relationship between PCSK9 and AD risk is uncertain.

NARC-1 was initially introduced as a molecule believed to support neuronal survival and regulate synaptic reorganization, suggesting an important role in both neurodevelopmental and neurodegenerative processes [58]. However, subsequent findings revealed that PCSK9 expression is not limited to the nervous system but is actually much higher in peripheral organs such as the liver, intestines, and kidneys. In these tissues, PCSK9 functions by controlling lipid metabolism through the lysosomal degradation of low-density lipoprotein receptors (LDLR), which shifted scientific attention toward its metabolic role, particularly in the liver [59,60].

Nevertheless, the fact that this molecule was first identified in neuronal tissue continues to raise questions about its potential dual role—as both a lipid regulator and a maintainer of neuronal homeostasis [60,61]. In recent years, interest in the neurobiological aspects of PCSK9 has resurfaced, with several studies investigating how its expression in the brain may contribute to pathological processes such as Aβ accumulation and neuroinflammatory responses [33,62]. Therefore, although PCSK9 is now predominantly recognized for its systemic metabolic function, its neuronal origin still offers valuable insight into its possibly overlooked involvement in AD pathology [6,50].

**Figure 2:**
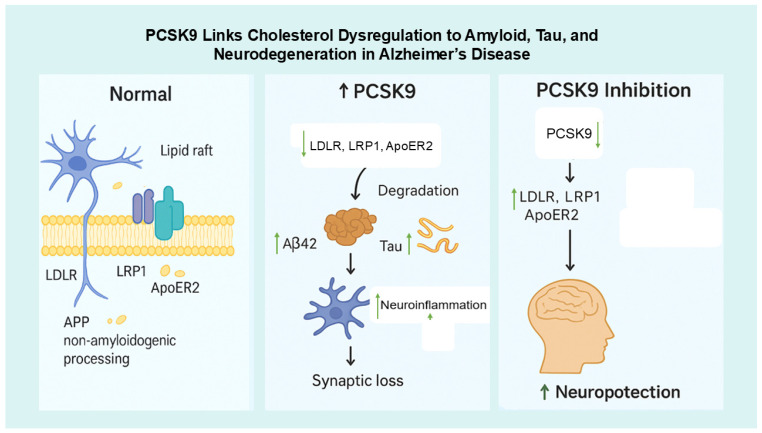
Proposed Mechanism Linking PCSK9 to AD Pathogenesis. ↓ = shows the reduced; ↑ = shows the increased/elevated. Under normal conditions, neuronal receptors such as LDLR, LRP1, and ApoER2 located in lipid rafts regulate cholesterol balance and promote non-amyloidogenic APP processing, preventing Aβ formation (left panel). When PCSK9 levels increase, these receptors are degraded, leading to higher Aβ42 accumulation, tau hyperphosphorylation, neuroinflammation, and synaptic loss, which contribute to neurodegeneration (right panel). Conversely, PCSK9 inhibition preserves LDLR, LRP1, and ApoER2, thereby improving cholesterol regulation, reducing Aβ and tau pathology, and promoting neuroprotection.

Mechanistically, PCSK9 may serve as a molecular intersection between cholesterol metabolism, neuronal apoptosis, and synaptic integrity—three central pathways implicated in AD. Through the degradation of LDLR and ApoER2, PCSK9 may disturb neuronal cholesterol balance, consequently affecting membrane dynamics and APP processing [6,61,62]. Concurrently, elevated PCSK9 levels have been linked to the activation of pro-apoptotic cascades and diminished neuronal survival, consistent with its early characterization as NARC-1, e.g., via downregulation of ApoER2, activation of JNK/c-Jun/caspase-3 pathways, and modulation of Bcl-2/Bax ratios [52,62,63]. These observations imply that the neuronal actions of PCSK9, though often eclipsed by its systemic metabolic roles, could represent a crucial molecular nexus connecting lipid dysregulation with neurodegenerative mechanisms in AD in Figure 2.

At a mechanistic level, PCSK9 appears to function as a critical hub where cholesterol metabolism, programmed cell death, and synaptic health converge [64]. This interconnected role is managed through its control over LDLR family receptors. When PCSK9 sends receptors like LDLR, LRP1, and ApoER2 for degradation, it directly hinders the neuron’s ability to take in cholesterol [65]. Since cholesterol is a vital building block for synaptic vesicles and maintaining membrane flexibility, this disruption weakens synaptic structure and function.

At the same time, the loss of the ApoER2 receptor has a separate grave consequence. This receptor is activated by Reelin, a neuroprotective signal. Without ApoER2, the crucial Dab1/PI3K/Akt survival pathway is shut down, leaving neurons susceptible to apoptosis and accelerating tau pathology. Therefore, with one primary action—degrading key receptors—PCSK9 manages to disrupt lipid balance, weaken synapses, and activate cell-death signals all at once. This positions it as a key integrator of several central pathways known to be involved in Alzheimer’s disease [6,52,61,62].

### 4.1. Clinical Studies

Findings from human research indicate that PCSK9 may play a role in Alzheimer’s pathology, particularly in relation to tau metabolism. Elevated levels of PCSK9 have been reported in cerebrospinal fluid and brain tissue from AD patients, often in parallel with increases in total tau or phosphorylated tau biomarkers [61]. These patterns suggest that PCSK9 could be involved in pathways linked to neurodegeneration [64].

However, when examined from a genetic perspective, the evidence appears much weaker. GWAS have not identified any direct link between PCSK9 variants and Alzheimer’s risk [25]. Similarly, Mendelian randomization studies—which test whether genetically determined changes in PCSK9 influence disease susceptibility—have generally suggested that PCSK9 is unlikely to be a causal determinant of AD [23].

The existing PCSK9 inhibitors, like evolocumab and alirocumab, are already proven champions at their main job: drastically lowering LDL cholesterol and cutting cardiovascular risk [66,67]. Importantly, extensive studies confirm they do this without any negative impact on cognitive function. Even more intriguing, some early, smaller studies hint that these drugs might actively support brain health, though we need more research to be sure [50]. Another critical point is that antibodies like alirocumab and evolocumab are large molecules that do not easily cross the healthy blood–brain barrier [68]. This suggests that any protective effects seen in laboratory studies probably occur through an indirect route. The benefits likely stem from improved systemic cholesterol levels and better overall vascular health, rather than from the drugs acting directly within the brain [11,27,50]. To conclusively determine if these medications can modify the course of Alzheimer’s, we need new clinical trials specifically focused on at-risk and affected populations. These studies, while establishing cognitive safety, deliberately excluded participants with major cognitive impairment or dementia. Consequently, it is unclear if these reassuring results apply to the AD population, whose compromised blood–brain barrier and other disease-related factors could create different risks.

The link between PCSK9 and Alzheimer’s is not one-size-fits-all. It seems to be influenced by individual factors like a person’s sex, genetic makeup (especially the APOE gene), and other health conditions like heart disease [27]. This means that although PCSK9 shows great promise as a biomarker, it is not yet reliable enough to be the sole indicator for predicting someone’s Alzheimer’s risk or trajectory.

### 4.2. In Vivo Studies

Animal studies provide more consistent results. In transgenic mouse models predisposed to Aβ or tau pathology, overexpression of PCSK9 accelerates plaque deposition, enhances tau phosphorylation, and leads to more pronounced impairments in synaptic plasticity and memory [21,33]. Beyond its direct influence on Aβ and tau, PCSK9 also affects systemic processes that impact brain health [7]. In models of hyperlipidemia or vascular disease, high PCSK9 levels exacerbate endothelial dysfunction, blood–brain barrier leakage, and neuroinflammation [31,69,70]. Suppression of PCSK9, on the other hand, reduces oxidative stress and helps preserve vascular integrity [71]. These findings suggest that the protective effects of PCSK9 inhibition may stem not only from changes in Aβ metabolism but also from improved vascular and metabolic support [72,73,74].

A vascular perspective, suppressing PCSK9, bolsters the health of blood vessels, offering a crucial neuroprotective effect against Alzheimer’s disease. Research in mice with high cholesterol demonstrates that inhibiting PCSK9 lowers oxidative stress in endothelial cells [71]. It achieves this by tamping down NADPH oxidase and boosting the availability of nitric oxide (NO) [75]. The consequences are a less leaky blood–brain barrier (BBB) and improved cerebral blood flow.

This vascular shielding involves several signaling changes, such as a decrease in pro-inflammatory adhesion molecules (e.g., VCAM-1) and the stimulation of endothelial NO synthase (eNOS) [76]. Ultimately, by fortifying the neurovascular unit and making the BBB less permeable, PCSK9 suppression achieves two key things: it helps clear Aβ peptides from the brain via perivascular drainage routes, and it guarantees that neurons receive a steady supply of oxygen and nutrients. This mechanism is how curbing PCSK9 activity provides protection against the vascular aspects of Alzheimer’s pathology [33,77].

### 4.3. In Vitro Studies

Cell culture experiments provide mechanistic detail that complements animal data. A recurring observation is that PCSK9 reduces the expression of lipoprotein receptors—including LDLR, LRP1, and ApoER2—on neurons and astrocytes [16,18]. Since these receptors are essential for cholesterol uptake and Aβ clearance, their downregulation favors extracellular Aβ accumulation [1].

Arya et al. (2025) [78] have identified a novel pathway through which PCSK9 may play a role in AD. This protein appears to affect the processing of Aβ precursor protein (APP), steering it toward a pathway that favors the production of harmful fragments. By enhancing the activity of certain enzymes, PCSK9 promotes the generation of the toxic Aβ42 peptide, the main component of plaques that impair brain function. In parallel, Bell et al. (2023) [17] reported that under cellular stress, the body tends to overproduce PCSK9. Excessive levels of this protein can trigger programmed cell death (apoptosis), and when this occurs in neurons, it leads to neuronal loss—a defining feature of neurodegenerative disorders.

While scientists are still piecing together PCSK9’s exact functions in the brain, current research suggests it helps control neuronal receptors that manage lipids, cell signaling, and survival. Laboratory studies show that PCSK9 downregulates crucial receptors, including LDLR, LRP1, and ApoER2 [16,18,37]. These receptors are vital for maintaining cholesterol balance and processing ApoE in the brain. Interfering with this lipid-signaling system plays a key role in the buildup of Aβ-amyloid. For instance, when PCSK9 degrades LRP1—a primary receptor for clearing Aβ—it hinders the removal of these peptides from the brain’s fluid.

At the same time, PCSK9 lowers the presence of LDLR and ApoER2 on the cell surface, leading to a cholesterol shortage inside neurons. This deficit changes the character of lipid rafts, which are specialized membrane zones that gather β- and γ-secretase enzymes. As a result of these changes, these rafts more readily promote the amyloidogenic breakdown of APP, boosting the generation of Aβ42. In summary, PCSK9 promotes Aβ pathology via a dual mechanism: it disrupts the clearance of Aβ while also accelerating its production, all by modulating central lipid signaling pathways [6,7].

Although not all studies fully agree on the molecular mechanisms, the majority point toward a scenario in which PCSK9 enhances amyloidogenic processing while simultaneously undermining neuronal survival [6].

### 4.4. Integrative Studies

A key challenge, however, is the blood–brain barrier, which limits the penetration of large monoclonal antibodies (alirocumab/evolocumab). Current PCSK9 inhibitors likely act indirectly via systemic lipid modulation rather than direct effects within the central nervous system [79,80]. Next-generation approaches, such as small molecules or RNA-based therapeutics, may overcome this barrier [51].

### 4.5. Future Priorities

Future research on PCSK9 in AD should include long-term studies that track. PCSK9 levels alongside core biomarkers to clarify its role in disease progression [26]. Genetic methods such as Mendelian randomization may help determine whether PCSK9 has a causal role or reflects secondary effects [46]. Since its impact may vary by sex, analyses comparing men and women are needed [15]. Randomized trials in high-risk groups, such as APOE ε4 carriers, could further test whether targeting PCSK9 influences biomarkers and cognition. The limited penetration of PCSK9 monoclonal antibodies (evolocumab) across the blood–brain barrier poses a principal challenge for their application in AD, likely preventing direct CNS effects. This suggests that their efficacy in models stems mainly from peripheral actions. A more promising therapeutic path involves developing next-generation PCSK9 inhibitors capable of reaching the brain. The results of forthcoming trials in Alzheimer’s populations will be essential to validate this strategy and inform future drug development.

### 4.6. Limitations

Our findings must be viewed in light of several constraints. The significant variation in the methodologies of the included studies prevented a pooled statistical analysis, necessitating a descriptive review. Another key point is the gap between robust animal data and the more ambiguous human genetic evidence, which has yet to confirm a causal relationship. Additionally, the recorded cognitive safety of PCSK9 inhibitors is based on trials focused on cardiovascular health that did not enroll cognitively impaired individuals, raising questions about its relevance for Alzheimer’s patients. A major pharmacological hurdle is that current versions of these drugs do not readily penetrate the blood–brain barrier, potentially restricting their direct action in the brain. While we have strived for a thorough examination of the literature, this area of science is advancing quickly, and the most recent discoveries may not be reflected here.

## 5. Conclusions

Preclinical models consistently show that PCSK9 promotes amyloidogenic processing, tau phosphorylation, and neuronal dysfunction, while PCSK9 inhibition often mitigates these effects and preserves cognition. Human studies are inconsistent: elevated PCSK9 in the brain or CSF has been linked to tau biomarkers in some cohorts, but large genetic and epidemiological analyses do not support a direct causal role. Existing PCSK9 inhibitors are cardiovascularly effective and cognitively safe, yet their impact on Alzheimer’s biomarkers has not been conclusively tested. Overall, PCSK9 is currently best viewed as a potential disease modifier or biomarker; targeted longitudinal studies and CNS-penetrant therapeutic trials are required to establish its clinical relevance in AD.
